# Determination of the target of monoclonal immunoglobulins: a novel diagnostic tool for individualized MGUS therapy, and prevention and therapy of smoldering and multiple myeloma

**DOI:** 10.3389/fimmu.2023.1253363

**Published:** 2023-10-31

**Authors:** Sylvie Hermouet, Edith Bigot-Corbel, Jean Harb

**Affiliations:** ^1^ Nantes Université, INSERM, Immunology and New Concepts in ImmunoTherapy, INCIT, UMR 1302, Nantes, France; ^2^ Laboratoire d’Hématologie, CHU Nantes, Nantes, France; ^3^ Laboratoire de Biochimie, CHU Nantes, Nantes, France

**Keywords:** monoclonal gammopathy of undetermined significance (MGUS), smoldering multiple myeloma (SMM), multiple myeloma (MM), monoclonal immunoglobulin, infectious pathogens, glucosylsphingosine (GlcSph), therapeutic targets, therapy

## Abstract

Subsets of patients diagnosed with a monoclonal gammopathy of undetermined significance (MGUS), smoldering multiple myeloma (SMM) or multiple myeloma (MM), present with a monoclonal immunoglobulin (Ig) specific for an infectious pathogen, including hepatitis C and B viruses (HCV, HBV), *Helicobacter pylori* and several *Herpesviruses*. Such cases are likely initiated by infection, since in the context of HCV- or HBV-infected patients, antiviral therapy can lead to the disappearance of antigenic stimulation, control of clonal plasma cells, and reduced or suppressed monoclonal Ig production. Complete remission has been obtained with anti-HCV therapy in refractory MM with a HCV-specific monoclonal Ig, and antiviral treatments significantly improved the probability of survival of MM patients infected with HCV or HBV prior to the diagnosis of MM. Monoclonal Igs may also target glucolipids, particularly glucosylsphingosine (GlcSph), and GlcSph-reducing therapy can lead to complete remission in SMM and MM patients presenting with a GlcSph-specific monoclonal Ig. The present review describes the importance of determining the target of the monoclonal Ig of MGUS, SMM and MM patients, and discusses the efficacy of target-reducing treatments in the management of MGUS, SMM and MM cases who present with a monoclonal Ig reactive against a treatable infectious pathogen or GlcSph.

## Introduction

Multiple myeloma (MM) is a highly malignant blood malignancy characterized by the expansion of a plasmacytic clone that produces large quantities of a single immunoglobulin (Ig), called monoclonal Ig. MM is preceded by asymptomatic monoclonal gammopathy of undetermined significance (MGUS) and/or smoldering myeloma (SMM) (1, 2). Over the years, a minority of MGUS evolves toward SMM and MM. Presently, it is not possible to predict the evolution of MGUS, and despite great advances in treatments, MM remains incurable. If screening for MGUS is becoming more frequent, in standard practice MGUS patients are simply monitored. SMM are more frequently studied, and treated, with the hope of suppressing plasmacytic clones when there are still minor (2–4). Indeed, being able to eliminate the plasmacytic clone at the MGUS or SMM stages would prevent evolution toward MM. However, so far most treatment strategies used at the SMM stage only delay progression toward MM (3, 4). More encouraging results have been obtained with 3-drug combination therapy: complete remission was achieved for 11/16 SMM patients, and these patients remained progression-free for at least 3 years (3).

Ideally, curative treatments should address and suppress the cause(s) of disease. Unfortunately, the causes of MGUS, SMM and MM have long remained unknown. Reasoning that most Igs are produced to counter infection, and that latent infection and subsequent inflammation are established causes of malignant transformation in solid cancers as well as in T-cell and B-cell lineage malignancies, latent infection seemed a possible cause of MGUS and subsequent evolution toward SMM and MM (5–7). Surprisingly since monoclonal Igs trigger many of MM symptoms and are major markers for the monitoring of clonal gammopathies, monoclonal Igs have long been neglected in research, diagnostics and therapy, and considered not to have any antibody function.

Yet it was possible that the targets of subsets of monoclonal Igs could be infectious pathogens. Initial pathogenic events leading to chronic lymphocytic leukaemia (CLL) and lymphoma include latent infection and chronic antigen stimulation and B-cell receptor stimulation from component of yeasts and fungi results in increased cell proliferation and survival (8–10). Similarly, chronic antigenic stimulation by antigens from infectious agents is a pathogenic mechanism leading to MGUS and MM (11–13). In particular, MM has been associated with viral infection, by hepatitis C virus (HCV), human immunodeficiency virus (HIV), or Epstein Barr virus (EBV) (14–18). In addition, a self-antigen, glucosylsphingosine (GlcSph), was reported to be the target of monoclonal Igs from MGUS and MM patients with or without Gaucher’s disease (19, 20).

This new pathogenic model of monoclonal gammopathies opened novel possibilities of prognosis and treatment. Indeed, knowing the target of a patient’s monoclonal Ig may help precise the prognosis in MM: patients with MM linked to GlcSph were reported to present with a mild form of MM disease, whereas patients with MM linked to EBV tended to have a more severe form of MM disease (17, 19–25). Regarding treatment, if the target of a patient’s monoclonal Ig can be suppressed, chronic antigen-stimulation should disappear, resulting in better control of the plasmacytic clone. The efficacy of such “target antigen reduction therapy” is now proven for monoclonal gammopathies, including MM, linked to GlcSph, HCV, and HBV (21–24). In HCV- or HBV-infected patients, treating the viral infection improved MGUS and MM disease and patient overall survival (21–24).

Importantly, these studies and results were made possible thanks to the development of several new assays based on the protein array technology, or derived from immunoblotting assays, which were specifically designed to identify the target of monoclonal Igs from patients. The aim of the present review is to describe these assays and how they can be useful to clinicians and their patients.

## Separation of monoclonal Igs from non-clonal Igs

MGUS and MM patients present with large amounts of monoclonal Ig, which can be detected in blood serum by serum protein electrophoresis (SPE), or mass spectrometry assays (26–31). Prior to the analysis of the specificity of recognition, monoclonal Igs must be purified, i.e. preparations of monoclonal Igs must be free from polyclonal Igs present in the blood serum of patients. Obviously this first step is required in MGUS but also in MM, even though the quantity of polyclonal Igs is often reduced in MM patients. A patient’s monoclonal IgG or IgA can be separated from non-clonal Igs using electric charge by running a SPE on an agarose gel, and after staining of one lane of the gel, by cutting carefully the band corresponding to the patient’s monoclonal IgG or IgA, then eluting the monoclonal Ig from the gel into phosphate buffer saline (PBS) (17, 18, 25, 32). The purity of the monoclonal IgG or IgA preparation can be verified using isoelectric focusing (IEF) followed by blotting and immune revelation (17, 18, 25, 32). Due to the much larger molecular mass of the monomers that constitute pentameric IgMs (185-190 kDa), compared to dimeric IgGs (150 kDa) and IgAs (160 kDa), this approach does not work as well for monoclonal IgMs.

## Identification of infectious targets of purified monoclonal Igs

### The multiplex infectious antigen array assay

**Figure 1:**
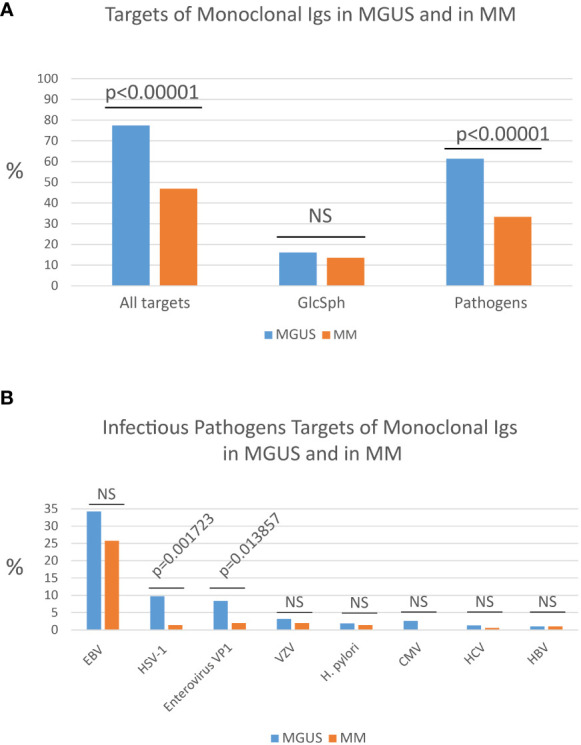
Percentages of MGUS and MM patients with a monoclonal Ig of known specificity. **(A)** Percentages (%) of patients with a monoclonal Ig that targets either an infectious pathogen or glucosylsphingosine (GlcSph) in cohorts of MGUS (n=155) or MM (n=147) patients. **(B)** Frequency of the different infectious targets of monoclonal Igs in MGUS and in MM: EBV, Epstein-Barr virus; HSV-1, herpes simplex virus 1; Enterovirus VP1 protein PALTAVETG or PALTAAETG sequences of human poliovirus type 1/3 and coxsackievirus type B1/B3; VZV, varicella zoster virus; *H*. *pylori*, *Helicobacter pylori*; CMV, cytomegalovirus; HCV, hepatitis C virus; HBV, hepatitis B virus. The Chi-2 test was used to compare the % of patients in the MGUS group and in the MM group. *P*<0.05 was considered significant. NS, not significant.

Following the reasoning that any infectious pathogen leading to the development of a MGUS or MM had to be associated with chronic, easily undiagnosed (latent) infection, a new test called the multiplex infectious antigen array (MIAA) assay was designed, that consists of recombinant antigens or proteins, peptides, and lysates from viruses, bacteria and parasites associated with chronic, latent infection in humans. The MIAA assay tests for 10 pathogens, including seven viruses (EBV, Herpes simplex virus 1 (HSV-1), HSV-2, cytomegalovirus (CMV), varicella zoster virus (VZV), HBV, HCV), one bacterium [*Helicobacter pylori* (*H. pylori*)], and two parasites [*Toxoplasma gondii* (*T. gondii)*, *Borrelia burgdorferi* (*B. burgdorferi*)] (17, 18, 25, 32, 33). Infectious lysates, proteins, peptides and antigens are spotted in triplicate on glass slides with 16 pads of nitrocellulose (33). The assay is performed with 4 µg purified monoclonal IgG or IgA, which are incubated on MIAA slides at room temperature. After washing, MIAA slides are incubated with fluorescence-labeled goat secondary antibodies against either human IgG (H+L) or human IgA (Fc), and fluorescence signals are detected using an imaging system scanner and quantified (17, 18, 25, 32, 33). Five fluorescence thresholds of specific positivity are used: 500, for HCV, *H. pylori*, *T. gondii*; 1,000, for HSV-1 and HSV-2; 1,200, for CMV; 1,400, for EBV and VZV; and 1,800 for *B. burgdorferi* (17, 18, 25). Fluorescent signals below the thresholds are considered negative. The MIAA assay has been used to analyse the specificity of recognition of purified monoclonal Igs from hundreds of patients: ~50% monoclonal Igs were found to target one of seven infectious pathogens (**Figure 1A**) (17, 18, 25, 32–35, and unpublished data). The seven infectious agents thus associated with MGUS and MM are, by order of frequency: EBV, HSV-1, VZV, *H. pylori*, CMV, HCV and HBV (**Figure 1B**) (17, 18, 25, 32–35).

### Other infectious protein/peptide microarrays

Other microarrays may be used to determine the eventual infectious target of purified monoclonal Igs. For instance, the PEPperCHIP*®* Infectious Epitope Micro-Arrays (PEPperPRINT Gmbh, Heidelberg, Germany) allowed to identify sequences shared by the VP1 coat proteins of human Enteroviruses *–* notably poliovirus type 1 and type 3 and coxsackievirus B1 and B3 – and specifically recognized by purified monoclonal Igs (35). These microarrays cover several thousands linear B-cell epitopes, translated into thousands of peptides, from 196 pathogens known to cause infection in humans. These assays typically require ≥30 µg purified monoclonal Ig. The two Enterovirus VP1 coat protein sequences identified as monoclonal Ig targets, PALTAVETG and PALTAAETG, were reported to be recognized by monoclonal Igs from 8.4% MGUS patients and 2.0% MM patients (**Figure 1B**) (34, 35).

Knowing which pathogen is the target of a patient’s monoclonal Ig may become important in terms of prognosis. Indeed, certain pathogens, such as CMV, were not found among the targets of monoclonal Igs from MM patients (17, 25). Similarly, HSV-1 and Enterovirus VP1 are significantly less frequently targeted by monoclonal Ig in MM compared to MGUS (**Figure 1B**). Thus, MGUS initiated by HSV-1 or Enterovirus may be relatively unlikely to progress toward MM.

Knowing the target of a patient’s monoclonal Ig is also interesting in terms of treatment: for MGUS and MM patients whose monoclonal Ig specifically recognizes HCV or HCV, implying that the gammopathy was initiated by HCV or HBV in these patients, antiviral therapy has recently been proven beneficial in terms of remission and improved survival (22–24).

## Identification of auto-antigen targets of monoclonal Igs

Population-based studies have revealed that a personal history of autoimmune disease significantly increased the risk of MGUS (36). Logically, subsets of monoclonal Igs have been reported to target auto-antigens from human proteins or glucolipids (19–21, 37–45). Information about the autoantigens targeted by monoclonal Igs has been obtained from studies of rare symptomatic monoclonal gammopathies, called monoclonal gammopathies of clinical significance (MGCS) (46, 47). Indeed, certain monoclonal Igs can cause organ lesions through autoantibody activity, for instance against collagen IV in the context of bullous skin disease, or anti-phospholipase A2 receptor in the context of glomerular nephropathy (48–50). It is also established that in the context of polyneuropathy, the monoclonal IgM of patients may target myelin-associated glycoprotein (MAG), chondroitin, or a ganglioside (34, 42–45). In addition, GlcSph is the target of monoclonal Igs from ~15% of MGUS and MM patients with or without Gaucher’s disease (**Figure 1B**) (19–21, 25).

To determine whether a monoclonal Ig targets a human protein, commercial microarrays can be used to test purified monoclonal Igs against several hundreds of peptides derived from human proteins. Such assays require at least 30 µg purified monoclonal Ig.

To determine whether a monoclonal Ig targets GlcSph, immunoblotting assays can be used (19, 20, 25). In short, polyvinylidene di-fluoride (PVDF) membranes are saturated with GlcSph, rinsed then blocked with bovine serum albumin (BSA). SPE gels run previously with the serum and purified monoclonal Ig of patients are blotted onto the GlcSph-saturated membranes using diffusion blotting. After blocking with BSA, the membranes are incubated with horseradish peroxidase-conjugated secondary antibodies, washed and revealed using chemiluminescence (19, 20, 25).

Knowing that the monoclonal Ig of a patient targets GlcSph or another self-antigen could become important for prognosis and therapy, since the new treatments that are emerging for MGCS may be of interest in other monoclonal gammopathies linked to auto-antigens (46, 47, 51–53).

### Interest for prognosis

Knowing which pathogen is the target of a patient’s monoclonal Ig is of interest in terms of prognosis. For instance, CMV is not found among the targets of monoclonal Igs from MM patients, which implies that CMV-associated MGUS are unlikely to progress toward MM (17, 25). Similarly, monoclonal Igs that target HSV-1 or Enterovirus VP1 protein are respectively seven and four times more frequent in MGUS than in MM, a possible indication that MGUS linked to HSV-1 or Enterovirus VP1 rarely evolve toward MM (**Figure 1B**) (35). Interestingly, a low risk of progression to MM was noted in MGUS patients with a history of autoimmune disease, which may indicate that monoclonal gammopathies initiated by self-antigens may be of good prognosis (51). In MM, two studies reported that patients with MM linked to GlcSph present with a mild form of MM disease (20, 25). In contrast, MM patients with a monoclonal Ig specific for EBV nuclear protein 1 (EBNA-1) presented with a greater invasion of bone marrow by clonal plasma cells, a higher creatinin level, and a higher β_2_-microbulin level, hence tended to have a more severe form of disease (17, 25). Using a different approach, other groups have reported EBV infection to be associated with poor prognosis in MM patients (54). Prospective studies on large cohorts of patients are necessary to confirm these preliminary results.

### Interest for therapy

The first report of the efficacy of self-antigen target reducing therapy in patients was published by Nair et al. in 2020 (21). This report described two patients with Gaucher Disease (GD) and gammopathy (1 MGUS, 1 SMM), whose gammopathy was likely initiated by GlcSph since their monoclonal Ig reacted against GlcSph. For the two patients, GlcSph-reduction therapy with eliglustat, an oral inhibitor of glucosylceramide synthase, reduced the production of the monoclonal Ig. In a previous study in GD mice, GlcSph reduction therapy with eliglustat had been shown to prevent the development of both myeloma and B-cell lymphoma (55). Prospective studies of patients with GlcSph-reactive monoclonal Ig are needed to determine whether therapy to reduce GlcSph, prevents progression from MGUS to SMM and MM (21).

Regarding MGUS and MM associated with viruses, an interesting report published in 2013 by Panfilio et al. described the regression of MM disease with antiviral treatment in a patient with HCV infection (56). In this report, the antigen specificity of the patient’s monoclonal Ig was not studied, but a later study showed that ≥80% of monoclonal IgGs from HCV-infected MGUS and MM patients targeted a HCV protein (16). Logically, when antiviral therapy succeeded in eliminating HCV, i.e. when chronic antigen stimulation disappeared, reduction or suppression of clonal plasma cells and monoclonal Ig production were observed, and certain patients achieved complete remission of MM disease (22). Recent studies of two large cohorts of MM patients infected either with HCV or HBV prior to the diagnosis of MM, revealed that antiviral treatments significantly improved patient overall survival, demonstrating the importance of antiviral therapy in MM patients infected with HCV or HBV (23, 24). Again, prospective studies of MGUS or SM patients with HCV- or HBV-specific monoclonal Igs are needed to confirm that anti-viral therapy does prevent progression to MM.

Other viruses, notably EBV and other herpesviruses, appear to be able to initiate significant subsets of MGUS and MM (17, 25). It is well known that reactivations of HSV or VZV are frequent in MM patients during intensive chemotherapy, and reactivations can be treated using different antiviral drugs (57). Moreover, antiviral prophylaxis is advised in MM patients (58). Consequently, one can envision future prospective clinical studies that test the efficacy of anti-herpesvirus therapy on those MGUS or MM patients who produce a monoclonal Ig which specifically targets a herpesvirus (EBV, CMV, HSV-1, VZV).

Concerning bacterial infection, so far *H. pylori* is the only bacterium associated with the development of MGUS and MM (17, 25). The efficacy of anti-*H. pylori* antibiotic therapy has been tested in small cohorts of MGUS patients, with discordant results. Some authors reported that antibiotic treatment of *H. pylori* infection led to the disappearance of the monoclonal Ig and resolution of the gammopathy (59). For other authors, the monoclonal Ig persisted after patients were treated for *H. pylori* infection (60). Beside their small size (30-60 patients), the limits of these studies include the lack of proof of complete *H. pylori* eradication, and lack of evidence that MGUS disease was initiated by *H. pylori* since the target of monoclonal Igs was not investigated (59, 60). Recent studies estimate that while 25-30% individuals are infected by *H. pylori*, only ~2% of monoclonal Igs target *H. pylori* (17, 25). Thus, the efficacy of anti-*H. pylori* antibiotic therapy should be tested on those MGUS or MM patients who produce a monoclonal Ig proven to specifically recognize *H. pylori*.

## Discussion

Because MM is still incurable, it is worth trying to prevent MM by treating patients at the MGUS stage, especially since many patients remain for years at the MGUS stage. Most MGUS being asymptomatic, chemotherapy is typically not an option at the MGUS stage. Recently it was demonstrated that a majority of MGUS cases (>70%) appear to be initiated by an infectious pathogen (≥60%), or by GlcSph (~15%). In contrast, at the MM stage the target of only ~40% of monoclonal Igs is an infectious pathogen. Efficient drugs exist that counter many infectious pathogens, or GlcSph, which suggests that “target-reducing” treatments should be tested at the MGUS stage, in patients for whom one can identify the target of the monoclonal Ig (i.e., probable disease-initiating event). This novel approach to the management of gammopathies has been proven successful for monoclonal gammopathies driven by GlcSph, HBV or HCV (21–24).

**Figure 2:**
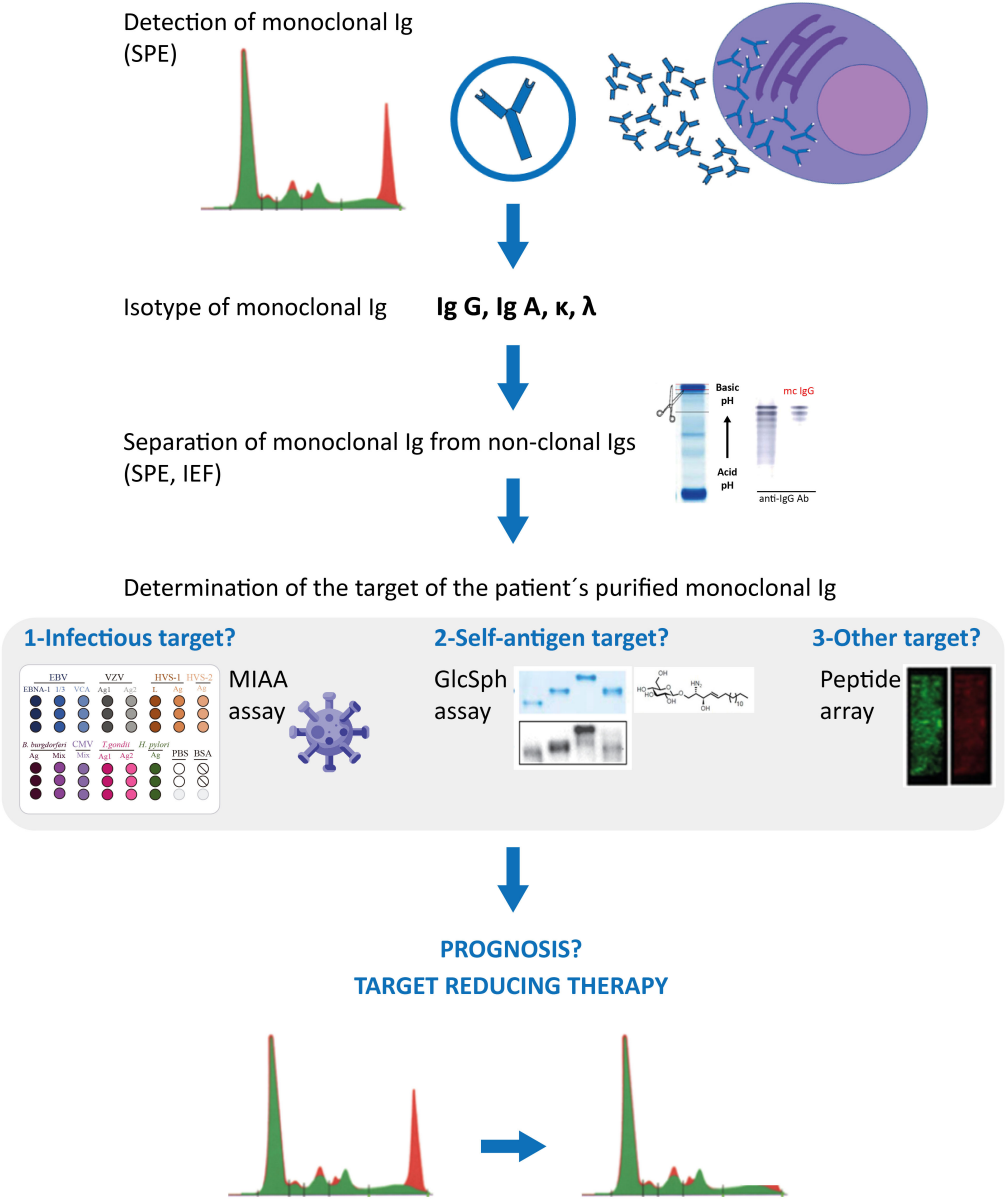
Characterization of monoclonal Igs from MGUS and MM patients. Clonal plasma cells of MGUS and MM patients secrete large amounts of a single Ig (= monoclonal Ig), which can be detected in blood serum by serum protein electrophoresis (SPE) (red peak); mass spectrometry assays can also be used to detect monoclonal immunoglobulins. Characterization of the monoclonal Ig include its quantification (most often by a immuno-turbidimetric method; more rarely by immuno-nephelemetric method or by orthogonal quantification of the peak) and identification of the Ig’s heavy (γ, α, μ, δ, ε) and light (κ λ) chains by immunofixation. Monoclonal IgGs or IgAs can be separated from non-clonal Igs in serum by SPE, elution from the SPE gel, and the degree of purity of the monoclonal Ig preparation can be verified by isoelectric focusing (IEF), western blotting and immunorevelation, as published (17, 18, 24, 31). The target of the purified monoclonal Ig can be determined using the multiplex infectious antigen microarray (MIAA) assay, which tests for the 7 most frequent infectious pathogen targets identified so far (17, 18, 24, 31), then with an assay that tests for GlcSph (lyso-glucosylceramide, LGL-1) (19–21). These assays require <5 μg purified monoclonal Ig and allow the identification of the target of >70% monoclonal IgG/A of MGUS patients, and >40% monoclonal IgG/A of MM patients. Purified monoclonal Igs remaining without an identified target after the MIAA and GlcSph assays may be analysed with microarrays that test for thousands of peptides; such arrays typically require ≥30 μg of purified monoclonal Ig. Knowing the target of a patient’s monoclonal Ig may provide information on the risk of transformation of MGUS into MM (seemingly low for MGUS associated with HSV-1 or Enterovirus VP1), on the prognosis of MM patients (good for GlcSph-initiated MM, more severe for EBV-initiated MM), and allow to propose to patients “target reducing therapy”, already proven beneficial for MGUS and MM cases initiated by GlcSph, HCV or HBV, in terms of reduction or suppression of clonal plasma cells and monoclonal Ig production, as well as improved patient survival (21–23).

Antiviral treatment of HCV-infected patients should prevent the development of HCV-driven IgG or IgA MGUS, as well as the progression of HCV-initiated MGUS into MM (22–24). Regarding HBV, ~60% of monoclonal gammopathies diagnosed in HBV-infected patients appear to be initiated by HBV, thus it is important to determine whether the gammopathy of HBV-infected patients is driven by the virus, or not. That not all infected MGUS or MM patients have a pathogen-initiated gammopathy is not restricted to HBV: it is also true for herpesviruses, Enteroviruses, and *H. pylori*. Patients with infection-initiated MGUS or MM can now be identified by the determination of the target of the patients monoclonal Ig (G or A), thanks to novel assays that allow such analysis of monoclonal Igs: the GlcSph assays, the MIAA assay, other infectious peptide microarrays. For MGUS, SMM and most MM patients, it is necessary to carefully separate the monoclonal Ig from non-clonal Igs in blood serum before performing the assays that determine the target of monoclonal Igs. **Figure 2** summarizes the complete laboratory work-up necessary to identify the target of monoclonal Igs.

In conclusion, using the MIAA assay, other protein arrays, and the GlcSph assay, it is now possible to determine the target of the majority of monoclonal IgGs and IgAs. Preliminary studies indicate that the target of a patient’s monoclonal Ig reflects an early event of MGUS or MM disease, and may serve as a prognosis marker and new therapeutic target. Clearly, anti-viral therapy should be prescribed as early as possible in the development of clonal gammopathies linked to viruses, ideally at the MGUS stage, though benefits in terms of improved overall survival were also demonstrated at the MM stage (22–24). For patients with a monoclonal Ig reactive against a self-antigen, knowing that autoimmunity initiated the monoclonal gammopathy should help understand the immune environment that leads to MM and adapt treatment (61). Prospective studies in large cohorts of patients are necessary to validate the prognostic and therapeutic interest of each of the infectious or auto-antigen targets of monoclonal Igs. Already, target-reducing therapy has been shown to benefit MGUS and MM patients who present with a monoclonal Ig specific for GlcSph, HCV or HBV.

## Author contributions

SH: Writing – original draft, Writing – review & editing. EB-C: Writing – review & editing. JH: Writing – review & editing.
